# Feasibility of Serum Galectin-1 as a Diagnostic Biomarker for Metabolic Dysfunction-Associated Steatotic Liver Disease: A Study on a Segment of the Chinese Population Using Convenience Sampling

**DOI:** 10.3390/biomedicines13020425

**Published:** 2025-02-10

**Authors:** Ting Zeng, Fang Li, Min Yang, Yao Wu, Wei Cui, Huaming Mou, Xiaohe Luo

**Affiliations:** 1 Department of Laboratory Medicine, Chongqing University Three Gorges Hospital, School of Medicine, Chongqing University, Chongqing 404000, China; ztingcc@163.com (T.Z.); lifang12232022@163.com (F.L.); wu_yao22@163.com (Y.W.); 2 The Center of Clinical Research of Endocrinology and Metabolic Diseases in Chongqing, Chongqing University Three Gorges Hospital, School of Medicine, Chongqing University, Chongqing 404000, China; 3 Department of Cardiovascular Medicine, Chongqing University Three Gorges Hospital, School of Medicine, Chongqing University, Chongqing 404000, China

**Keywords:** metabolic dysfunction-associated steatotic liver disease, serum galectin-1 levels, diagnostic marker, fatty liver index, hepatic steatosis index

## Abstract

**Background/Objectives**: Metabolic Dysfunction-Associated Steatotic Liver Disease (MASLD) is commonly considered as a hepatic manifestation of metabolic syndrome, posing considerable public health and economic challenges due to its high prevalence. This study investigates the diagnostic potential of serum galectin-1 levels in MASLD patients. **Methods**: A total of 128 participants were analyzed for this study, comprising 68 healthy controls and 60 MASLD patients. The hepatic steatosis index (HSI) and fatty liver index (FLI) were calculated to evaluate the liver steatosis. Serum galectin-1 levels were measured using an enzyme-linked immunosorbent assay. We additionally conducted a comparative analysis of galectin-1 mRNA and protein expression levels in the liver tissue between the mouse models of MASLD, including ob/ob mice (*n* = 6), high-fat diet-fed C57 mice (*n* = 6), and the control group (*n* = 6). **Results**: Average serum galectin-1 levels significantly differed between groups, with lower values in the controls (*p* < 0.01). The frequency of MASLD increased with higher quartiles of galectin-1 levels (*p* < 0.01). The correlation analysis showed a positive relationship between serum galectin-1 and both HSI and FLI (*p* < 0.01). The multivariate logistic regression indicated that elevated galectin-1 was associated with an increased risk of MASLD (*p* < 0.01), yielding an area under the receiver operating characteristic curve for predicting MASLD at 0.745 (95% CI: 0.662–0.829). Hepatic galectin-1 levels were also elevated in the MASLD mouse model at both transcript and protein levels (*p* < 0.01). **Conclusions**: Serum galectin-1 can be used as a potential biomarker to help diagnose MASLD.

## 1. Introduction

Metabolic Dysfunction-Associated Steatotic Liver Disease (MASLD) was renamed from Nonalcoholic Fatty Liver Disease (NAFLD) at the 2023 European Association for the Study of the Liver (EASL) Congress [1]. The terminology has evolved to better reflect the disease’s metabolic nature. NAFLD was initially defined by the exclusion of alcohol-related causes [2]. In 2020, Metabolic-Associated Fatty Liver Disease (MAFLD) was introduced to emphasize metabolic factors such as obesity and diabetes, integrating these into the diagnostic criteria [3]. MASLD further refines this classification, encompassing a broader range of metabolic dysfunctions and liver-related conditions. These terms reflect a shift from exclusionary to more positive diagnostic criteria, highlighting the importance of metabolic factors in disease progression and management. Despite subtle differences, there is excellent congruence between NAFLD, MAFLD, and MASLD definitions, and affected patients usually meet the criteria for all [4].

MASLD has emerged as the predominant form of chronic liver disease [5], with a fast-growing global prevalence of approximately 30% worldwide [6], and it was predicted to reach 55.7%. It varies significantly across regions, as follows: Latin America (44.4%) and the Middle East (36.5%) show the highest prevalence, while the Asia–Pacific region (28.0%) and Western Europe (25.1%) have a relatively lower prevalence [7]. This increasing trend poses a significant challenge to public health systems.

The primary histopathological feature of MASLD is the accumulation of excessive lipids within hepatocytes [8]. More specifically, disorders in glucose and lipid metabolism can generate excess free fatty acids that enter liver cells and are converted into triglycerides. Triglyceride buildup in liver cells leads to the formation of lipid droplets and initiates MASLD [9]. Various risk factors, including a high-fat diet, lack of physical activity, and genetic or epigenetic influences, can collectively contribute to the development of MASLD [10]. MASLD represents a spectrum of liver pathology, ranging from asymptomatic steatosis to more severe conditions, such as steatohepatitis, fibrosis, cirrhosis, and even hepatocellular carcinoma [11,12]. Emerging research indicates that MASLD is a multi-system disorder [13], impacting systemic glucose and lipid metabolism, as well as various extrahepatic conditions such as chronic kidney disease [14], type 2 diabetes mellitus (T2DM) [15], and cardiovascular disease [16]. This complex interplay of factors contributes to a substantial public health challenge, imposing significant healthcare and economic burdens on individuals and society as a whole. Hence, the timely recognition and precise evaluation of the progression of MASLD are essential for determining the prognosis of the condition. 

The galectin family, recognized for its significant conservation among various species, comprises soluble proteins possessing at least one carbohydrate-binding domain facilitating interactions with specific glycoconjugate ligands, thereby enabling the cross-linking of oligosaccharides and fulfilling various biological functions in both normal and pathological processes [17,18]. To date, 15 types of mammalian galectins have been identified, with galectin-1 being the first member isolated. The gene for *galectin-1* is located on human chromosome 22, specifically, at NC_000022.11. This gene encodes a protein of approximately 14 kDa, comprising 135 amino acids (https://www.ncbi.nlm.nih.gov/, accessed on 10 January 2025). The protein galectin-1, known for its specificity for β-galactosides, displays varying levels of expression in different tissues, with the highest levels observed in subcutaneous adipose tissue, followed by the vasculature and the female reproductive system [19]. Its role has been implicated in tumor development [20], immune regulation [21], inflammatory response [22], tissue repair [23], and obesity [24]. Numerous reports have indicated that galectin-1 may play a significant role in key metabolic processes. Research has indicated that the inhibition of galectin-1 resulted in a decrease in adipogenesis and lipid accumulation in vitro, achieved through the downregulation of key adipogenic transcription factors C/EBPα and PPARγ [24]. Concurrently, there was an upregulation of proteins involved in energy expenditure and lipid catabolism, such as ATP5B, COXIV, HSL, and CPT1 [25]. Additionally, studies have shown that galectin-1 can regulate glucose metabolism by modulating pancreatic insulin secretion [26]. Galectin-1 overexpression accelerates HCC development by triggering EMT in HCC cells via the PI3K/AKT signaling cascade [27]. Furthermore, recent research has highlighted the potential of serum galectin-1 as a diagnostic biomarker for a range of conditions, such as early rheumatoid arthritis [28], fetal growth restriction [29], polycystic ovary syndrome [30], and T2DM [31].

This study aims to evaluate the diagnostic value of serum galectin-1 for MASLD, given its strong correlation with insulin resistance (IR) and diabetes mellitus (DM).

## 2. Materials and Methods

### 2.1. Subjects

**Figure 1:**
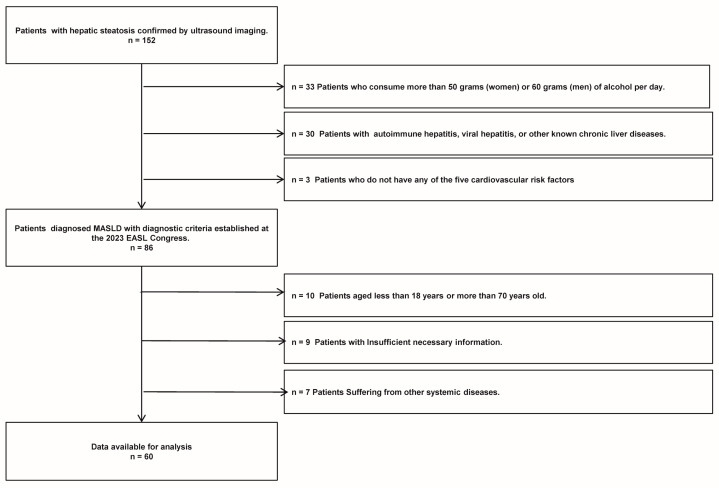
The MASLD patient flow chart of this study.

A total of 152 patients diagnosed with liver steatosis via ultrasound were selected from those who visited the Department of Endocrinology and Metabolism at Chongqing University Three Gorges Hospital, spanning the period from January 2020 to January 2021. Ultimately, 60 patients with MASLD who met the study criteria (including 27 males and 33 females) were included in the study. The specific flowchart of MASLD refers to Figure 1. The inclusion criteria for MASLD were the following: (1) confirmation of hepatic steatosis by an experienced ultrasound physician, (2) presence of one or more cardiometabolic risk factors (CMRF), and (3) age between 18 and 70 years old. The exclusion criteria for MASLD were as follows: (1) alcohol consumption exceeding 50 g per day for women or 60 g per day for men, (2) presence of autoimmune hepatitis, viral hepatitis, or other known chronic liver diseases, (3) insufficient necessary information, and (4) presence of other systemic diseases. Additionally, 68 healthy controls (including 33 males and 35 females), who underwent routine physical examinations at the same institution’s health check-up center during the same timeframe, were included. These controls, confirmed to have no liver steatosis or any other diseases, were matched with the patient cohort based on age and gender for participation in the study.

In addition, our investigation also queried the Gene Expression Omnibus (GEO) (https://www.ncbi.nlm.nih.gov/geo/, accessed on 23 June 2024) database for differentially expressed genes associated with MASLD in human samples. The data originated from the GEO database (GSE185051), and the samples comprised liver biopsy tissues from pediatric patients with MASLD (*n* = 51) and pediatric healthy controls (*n* = 4). Through high-throughput RNA sequencing, the transcriptomic changes associated with MASLD were analyzed. The screening thresholds applied in this study were a *p*-value < 0.05 and |fold change (FC)| > 2 [32]. In this study, we used the GEO2R to perform differential expression analysis and identify Differentially Expressed Genes (DEGs) between MASLD and control samples. Volcano was plotted using https://www.bioinformatics.com.cn (last accessed on 10 October 2024), an online platform for data analysis and visualization.

### 2.2. Clinical Data and Galectin-1 Measurement

The demographic characteristics of the participants, such as gender and age, were collected from their official identification documents. Anthropometric assessments, including blood pressure, height, weight, waist circumference (WC), and hip circumference (HC), were performed by qualified personnel. The body mass index (BMI) was computed using the formula body weight (kg)/height (m^2^), while the waist-to-hip ratio (WHR) was calculated as the ratio of WC to HC. Prior to the experiment, participants were instructed to adhere to their usual dietary habits, maintain consistent levels of physical activity, avoid consuming drugs and alcohol, and refrain from eating after 8 PM the day before. On the day of the experiment, fasting venous blood samples were obtained and promptly sent to the Medical Laboratory Department of the Three Gorges Hospital Affiliated to Chongqing University for detection and analysis with the Roche Cobas 775 analyzer. The detected indicators included the following: lipid profile—total cholesterol (TC), high-density lipoprotein cholesterol (HDL-C), low-density lipoprotein cholesterol (LDL-C), triglycerides (TG), and lipoprotein(a) [Lp(a)]; liver function—total protein (TP), albumin (Alb), globulin (Glb), γ-glutamyl transpeptidase (GGT), alanine aminotransferase (ALT), aspartate aminotransferase (AST), alkaline phosphatase (ALP), total bile acid (TB), direct bile acids (DB), and indirect bile acids (IDB). Additionally, the atherosclerotic index (AI), fatty liver index (FLI), and hepatic steatosis index (HSI) were calculated using the established formulas [33,34].

$$AI=\frac{TC-HDL}{HDL}$$

$$FLI=\frac{e^{0.953\;\times \;\log_{e}TG+0.139\times BMI+0.718\times \log_{e}GGT+0.053\times WC-15.745}}{1+e^{0.953\times \log_{e}TG}+0.139\times BMI+0.718\times \log_{e}GGT+0.053\times WC-15.745}\times 100$$

$$HSI=8\times \frac{ALT}{AST}+BMI(+2,\;if\;famale;+2,\;if\;diabetes\;mellitus)$$

Fasting venous blood samples were collected for serum isolation and stored at −80°C before analysis. The galectin-1 level in serum was detected using the galectin-1 enzyme-linked immunosorbent assay kit (Code No. EH203RB, Thermo Fisher Scientific, Waltham, MA, USA), with strict adherence to the manufacturer’s instructions. Liver ultrasonography was conducted on all participants by a skilled sonographer utilizing the Mindray Resona R7 (5.0 MHz transducer, SC6-1U). Subsequently, all reports were thoroughly reviewed by senior physicians.

### 2.3. Statistical Analyses

The SPSSAU project (Version 24.0, QingSi Technology Ltd., Beijing, China) [Online Application Software], retrieved from https://www.spssau.com, accessed on 24 February 2024, was employed for data analysis. A normal distribution test was initially conducted on the complete dataset and within distinct groups. Measurement data adhering to a normal distribution were expressed as the mean ± standard deviation, with a t-test utilized to evaluate differences between groups. When the distribution was not normal, the median (Q1, Q3) was used for the description, and comparisons were made using the Mann–Whitney U test. Categorical data were presented as frequencies (percentages) and assessed for group differences using the Chi-square test. According to the quartile levels of serum galectin-1, the subjects were stratified into four groups (Q1, Q2, Q3, and Q4). The Cochran–Armitage trend test was employed to determine the presence of any significant trends. The study utilized Pearson’s correlation test for normally distributed data and Spearman’s test for non-normally distributed data. Using a multivariate linear regression framework, the study conducted a comprehensive analysis to explore the potential correlations between serum galectin-1 levels and FLI/HSI, to investigate the relationship between serum galectin-1 levels and the presence of MASLD. The study utilized regression analysis to examine the relationship between serum galectin-1 levels as the independent variable and the dependent variables FLI and HSI. The independent variables included in the binary logistic regression analysis were HSI, FLI, AI, TG, ALP, and galectin-1. The dependent variable was the presence or absence of MASLD. A stepwise method was employed to screen the independent variables. Three models were developed for each analysis, controlling for confounding factors including age, sex, ALT, AST, TC, WHR, ALP, GGT, and TG. Logistic regression was employed to investigate the impact of galectin-1 on MASLD, and a receiver operating characteristic (ROC) curve analysis was conducted to assess its predictive ability in diagnosing MASLD. Statistical analyses were conducted using a two-tailed approach with a significance threshold set at *p* < 0.05.

### 2.4. Animal Experiments

In this study, we utilized specific-pathogen-free (SPF) C57BL/6J mice, procured from Guangzhou Saiye Biotechnology Co., Ltd. (Guangzhou, China), and leptin-deficient ob/ob mice, sourced from Changzhou Cavens Laboratory Animal Co., Ltd. (Changzhou, China). The experimental diets, including a normal control diet (NCD) and a 60% high-fat diet (HFD), were obtained from Nantong Telofei Feed Technology Co., Ltd. (Nantong, China). SPF-grade, irradiation-sterilized maintenance feed was supplied by Jiangsu Sye Pharmaceutical Biological Engineering Co., Ltd. (Changzhou, China).

Twelve 8-week-old male C57BL/6J mice were acclimated for one week prior to randomization into two groups as follows: one group received the NCD (Controls, *n* = 6), while the other was administered the HFD for 12 weeks (HFD, *n* = 6) [35,36]. Concurrently, 8-week-old male ob/ob mice were maintained on a standard diet (ob/ob, *n* = 6) [37]. To ensure a controlled experimental environment, the animal housing conditions were meticulously controlled, with the following parameters: temperature at 22 °C ± 2 °C, relative humidity at 50 ± 10%, and a 12 h light/dark cycle. The bedding material was replaced biweekly, and cages were subjected to autoclaving post-cleaning to ensure sterility. The mice were euthanized by cervical dislocation. The mouse was positioned on a textured surface. The experiment operator’s left thumb and index finger were employed to press down on the mouse’s head and neck while the right hand grasped the root of the mouse’s tail and pulled it back and upward, ensuring a rapid and humane euthanasia. After alcohol was sprayed, the mouse skin was cut along the midabdominal line and the liver tissue was removed and promptly stored in liquid nitrogen for subsequent experiments. Protease inhibitors and phosphatase inhibitors were added to RIPA buffer to prepare protein lysates for protein extraction. In accordance with the protocols outlined in the bicinchoninic acid protein assay kit (Code No. P0012, Beyotime Biotechnology, Shanghai, China), the quantification of protein concentration in the samples was effectively carried out. Subsequent to determining the optimal loading quantity, the proteins were fractionated on a 15% polyacrylamide gel and then transferred onto a 0.2 μm PVDF membrane. The membranes were incubated at 4 °C with gentle agitation on a shaker using antibodies targeting galectin-1 (Code No. sc-166618, 1:500, Santa, Dallas, TX, USA) and β-actin (Code No. ZB15001-HRP-100, 1:1000, Servicebio, Wuhan, China) overnight, after sealing with rapid sealant liquid. Subsequently, they were incubated with a horseradish peroxidase-conjugated goat anti-mouse antibody (Code No. GB23301, 1:10,000, Servicebio, Wuhan, China) for 1 h at room temperature. Following incubation, the membranes were treated with enhanced chemiluminescence detection solution and exposed using a chemiluminescence imaging system (ChemStudio SA2, Jena, Germany). RNA was isolated from mouse liver tissues utilizing Trizol (Code No. 9109, Takara, Tokyo, Japan). Subsequently, cDNA synthesis was performed using an RNA reverse transcription kit (Code No. RR047A, Takara, Tokyo, Japan). The synthesized cDNA was then combined with the quantitative polymerase chain reaction (qPCR) reagents (Code No. RR820A, Takara, Tokyo, Japan) to prepare the reaction mixture, which was subsequently subjected to amplification using a qPCR instrument (Analytik Jena AG, Jena, Germany). The thermal cycling conditions were set as follows: an initial denaturation step at 95 °C for 3 s, followed by 40 cycles of denaturation at 95 °C for 3 s, and annealing and extension at 60 °C for 30 s. The entire procedure was meticulously conducted in accordance with the manufacturer’s protocol. *β-actin* served as a stable endogenous control for normalization in the quantification of relative mRNA expression levels of *galectin-1*, employing the ∆∆Ct method. The primers utilized were as follows: *galectin-1* (forward primer: 5′-GCCTACACTTCAATCCTCGCT-3′; reverse primer: 5′-GTTCCCGGTGTTCGGTTCC-3′) and *β-actin* (forward primer: 5′-GCGAGCACAGCTTCTT-3′; reverse primer: 5′-TGACACGTGTTC-3′).

## 3. Results

### 3.1. Characteristics of Participants

**Figure 2:**
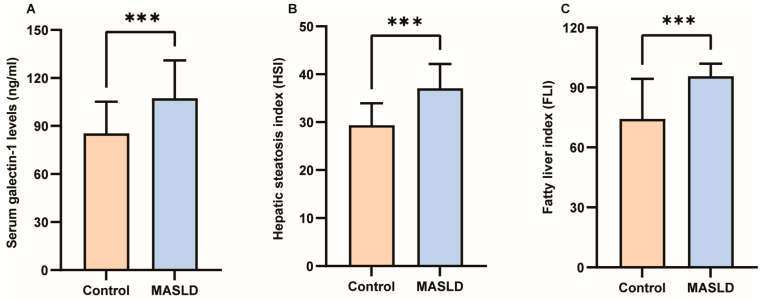
Differences in serum galectin-1, HSI and FLI between control and MASLD. (**A**) Serum galectin-1 differences between control and MASLD. (**B**) HSI differences between control and MASLD. (**C**) FLI differences between control and MASLD. Data represent the means ± SEM; *** *p* < 0.001. MASLD, metabolic-associated steatotic liver disease; HSI, hepatic steatosis index; FLI, fatty liver index.

The results of the Kolmogorov–Smirnov normality test for all measurement data are presented in Supplementary Table S1. Detailed clinical characteristics of the study participants can be found in Table 1. No significant differences were observed in gender, age, height, TC, lp(a), TP, Alb, or Glb between the two groups (*p* > 0.05). However, galectin-1, weight, BMI, WC, HC, WHR, TG, AI, HDL-C, LDL-C, AST, ALT, GGT, ALP, TB, DB, IDB, HSI, and FLI exhibited significant differences between the two groups (*p* < 0.05; *p* < 0.01; *p* < 0.001). As depicted in Figure 2A, the average value of galectin-1 was significantly lower in the control group (85.23 ng/mL) compared to the MASLD group (107.30 ng/mL). Furthermore, in individuals with MASLD, levels of HSI were considerably higher than those without MASLD (37.05 ± 5.10 vs. 29.35 ± 4.58, *p* < 0.001) (Figure 2B). Additionally, FLI levels were higher in individuals with MASLD than those without MASLD (95.66 ± 6.26 vs. 74.29 ± 20.08, *p* < 0.001) (Figure 2C). In the GSE185051 cohort, comparative liver transcriptome analysis between the MASLD group and the healthy control group identified 4994 genes as upregulated and 5283 genes as downregulated. Additionally, 13,897 genes demonstrated no significant differential expression [38]. Among them, galectin-1 was significantly upregulated (Supplementary Figure S1).

### 3.2. The Trend of MASLD in Increasing Galectin-1 Level Groups

As the galectin-1 level increased, there was a corresponding increase in the frequency of MASLD at 15.6%, 40.6%, 62.5%, and 68.8%, respectively, across each quartile group, and the trend of increasing frequency of MASLD across quartiles was found to be statistically significant (*p* < 0.01) (Table 2).

### 3.3. Analysis of the Correlation Between Serum Galectin-1 Levels and Other Metabolic Indicators

**Figure 3:**
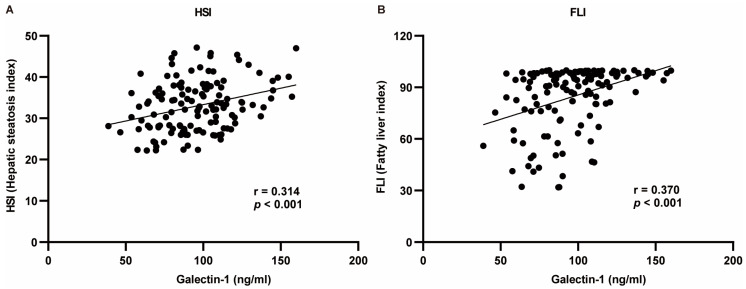
Regression analysis between HSI or FLI and serum galectin-1 levels using Pearson’s correlation analysis. (**A**) Galectin-1 was positively correlated with HSI. (**B**) Galectin-1 was positively correlated with FLI. r: regression coefficient. HSI, hepatic steatosis index; FLI, fatty liver index.

The indicators exhibiting significant differences between the two groups were selected for correlation analysis with galectin-1. The Pearson test was employed for indicators conforming to a normal distribution, while the Spearman test was utilized for those not conforming to the normal distribution criteria. The correlation analysis revealed that serum galectin-1 levels exhibited a negative correlation with HDL-C, TB, DB, and IDB and a positive correlation with weight, BMI, WC, HC, WHR, TG, AI, LDL-C, ALT, GGT, HSI, and FLI (*p* < 0.05, *p* < 0.01). No significant correlations were observed between AST or ALP and serum galectin-1 levels (*p* > 0.05) (Table 3). Given the positive association of galectin-1 levels with HSI as well as FLI, multivariate linear analysis was conducted to further explore this relationship (Figure 3).

Serum galectin-1 levels exhibited a positive correlation with FLI in the crude model (standardized β = 0.370, *p* < 0.001). After adjusting for age and gender in model I^a^, the association between serum galectin-1 level and FLI remained significant (standardized β = 0.362, *p* < 0.001). Similarly, after further adjustment for TC, ALT, AST, and ALP in model II^a^, the association between serum galectin-1 level and FLI remained significant (standardized β = 0.268, *p* < 0.001). In terms of the HSI analysis, there was also a positive correlation observed between serum galectin-1 level and the HSI in the crude model (standardized β = 0.314, *p* < 0.001). After adjusting for age and WHR in model I^b^, the association between serum galectin-1 level and HSI remained significant (standardized β = 0.228, *p* = 0.002). Furthermore, even after additional adjustments were made for age, WHR, TC, ALP, GGT, and TG in Model II^b^, the relationship between serum galectin-1 levels and his was still shown to be significant (standardized β = 0.218, *p* = 0.003) (Table 4). The diagnostics of the multivariable regression models for the FLI (Supplementary Table S2.1) and HSI (Supplementary Table S2.2) have been proven to be reasonable.

### 3.4. The Clinical Value of Galectin-1 Levels in Predicting MASLD

After the automatic identification of the model, the HSI, FLI, and galectin-1 remained significant predictors in the final model. According to the provided Table 5, these three variables accounted for 0.49 of the variations in MASLD occurrence. The derived model formula is as follows:

$$\ln\frac{p}{1-p}=-18.747+0.211\times HSI+0.082\times FLI+0.045\times galectin1$$

where *p* represents the probability of MASLD, and 1 − *p* represents the probability of the control. The final analysis revealed that HSI had a regression coefficient of 0.211 with a significant positive effect on MASLD (z = 3.068, *p* = 0.002), indicating an odds ratio (OR) of 1.234 which implies that a one unit increase in HSI is associated with a 1.234-fold change in MASLD occurrence. Similarly, FLI showed a regression coefficient of 0.082 with a significant positive effect on MASLD (z = 2.364, *p* = 0.018), resulting in an odds ratio (OR) of 1.085. Furthermore, the regression coefficient of serum galectin-1 level was 0.045, indicating a statistically significant positive effect on the occurrence of MASLD (z = 3.158, *p* = 0.002). The odds ratio (OR) was 1.046, suggesting that for every unit increase in serum galectin-1 level, there is a 1.046-fold increase in the likelihood of developing MASLD.

**Figure 4:**
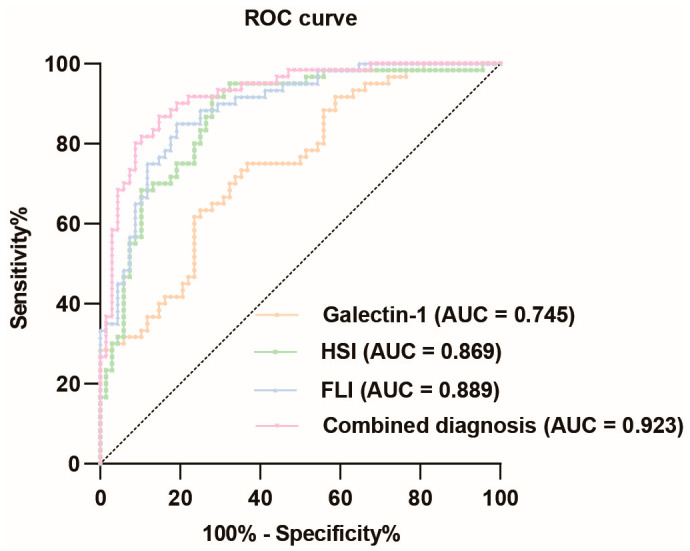
ROC curve analysis of galectin-1, HSI, FLI, and their combination diagnosis prediction of MASLD. The black dotted line represents the random classifier. The orange line, green line, blue line, and pink line represent the ROC curve of galectin-1, HSI, FLI, and their combination, respectively. The points above the black dotted line indicate a better probability than the random one. MASLD, metabolic dysfunction-associated steatotic liver disease; HSI, hepatic steatosis index; FLI, fatty liver index. AUC, area under the curve; ROC, receiver operating characteristic.

The ROC curve analysis of galectin-1 levels in predicting MASLD demonstrated a relatively high diagnostic value, with an AUC value of 0.745 (95% CI: 0.662–0.829). The optimal cut-off value was determined to be 0.383, resulting in a sensitivity of 0.633 and specificity of 0.750, corresponding to a concentration of 99.249 ng/mL for galectin-1. The HSI also exhibited a relatively high diagnostic value for MASLD, with an AUC value of 0.869 (95% CI: 0.806–0.932). The optimal cut-off point was determined to be 0.637, yielding a sensitivity of 0.917 and specificity of 0.721. Similarly, the FLI had a relatively high diagnostic value for MASLD with an AUC value of 0.889 (95% CI: 0.833–0.944). The optimal cut-off point was determined to be 0.659, yielding a sensitivity of 0.850 and specificity of 0.809. When these three indicators were combined, the corresponding AUC value increased to 0.923 (95% CI: 0.877–0.969). In summary, the ROC curve analysis revealed that galectin-1, HSI, and FLI each possessed significant diagnostic values for MASLD individually (Figure 4, Supplementary Table S3.1). To further validate the diagnostic value of the combined indicators, we conducted pairwise comparisons of the AUCs using the Delong test. The results indicate that the combined model of galectin-1, HSI, and FLI significantly outperforms galectin-1 and HSI individually in terms of diagnostic accuracy for MASLD, as evidenced by the higher AUC values and the statistical significance of the differences (*p* < 0.05). However, the difference between the AUC of FLI and the combined model is not statistically significant (*p* > 0.05) (Supplementary Table S3.2).

### 3.5. Increased Galectin-1 Expression in the Liver of MASLD Mice

**Figure 5:**
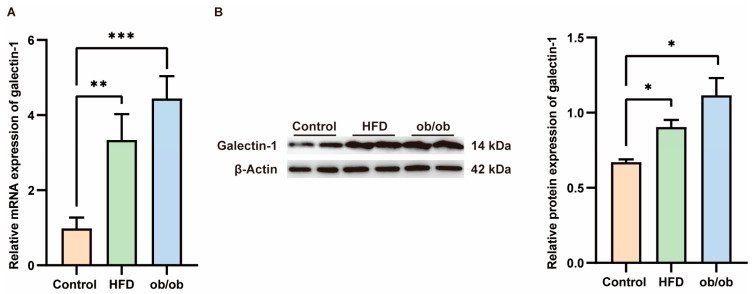
Increased galectin-1 expression in the liver of mice fed with a high fat diet (HFD) and obese mice. (**A**) Comparison of galectin-1 mRNA levels in livers of controls (*n* = 6) and HFD fed mice (*n* = 6) and ob/ob mice (*n* = 6). (**B**) Representative Western blots and relative protein expression of total liver lysates from controls (*n* = 6) and mice fed with HFD (*n* = 6) and ob/ob mice (*n* = 6). Data represent the means ± SEM; * *p* < 0.05, ** *p* < 0.01, *** *p* < 0.001.

Serum levels of galectin-1 are elevated in patients with MASLD, prompting us to investigate whether hepatic galectin-1 expression is altered in obese mice. Liver tissues were collected from ob/ob mice and mice fed a high-fat diet for 12 weeks. Successful model establishment was histologically validated through hematoxylin and eosin (HE) staining (Supplementary Figure S2). As confirmed by qPCR and Western blot analyses (Figure 5), both galectin-1 mRNA and protein levels exhibited an increase in the livers of ob/ob mice as well as HFD-fed mice compared to the normal control group (*p* < 0.01, *p* < 0.01, *p* < 0.05).

## 4. Discussion

Current research on MASLD focuses on the development of non-invasive diagnostic techniques, aiming to replace invasive procedures such as liver biopsies with serum assays or imaging modalities. It was found that serum galectin-1 levels were significantly elevated in individuals with MASLD. The correlation analysis demonstrated that serum galectin-1 levels were positively correlated with the FLI and HSI. After adjusting for gender, age, BMI, and additional factors, galectin-1 levels remained positively correlated with FLI and HSI. The results of the logistic regression analysis indicated that galectin-1 was a risk factor for MASLD. The ROC curve analysis demonstrated that the AUC value of galectin-1 levels was 0.745, suggesting that it may be a useful biomarker for predicting the occurrence of MASLD.

In discussing MASLD, it is essential to highlight its distinctions from MAFLD. Both conditions are diagnosed based on metabolic risk factors. Specifically, MASLD requires the presence of at least one out of five metabolic factors, whereas MAFLD is characterized by the presence of type 2 diabetes, overweight/obesity, or at least two additional metabolic factors. Research conducted by Terry Cheuk-Fung Yip and his colleagues indicates that, in both the general population and in hospital settings, fewer patients with NAFLD are reclassified under the MASLD criteria compared to the MAFLD criteria. The distinctions between MASLD and NAFLD are minimal, suggesting that findings from prior NAFLD-related research likely remain applicable under the revised MASLD classification [39].

MASLD is a disease characterized by the abnormal accumulation of lipids in the liver as the initial factor and the key pathological feature. The initial stage of MASLD is metabolic dysfunction-associated steatotic liver, which is a benign lesion. It can be restored to a healthy state by improving the diet and undertaking moderate exercise. MASLD can progress to the stage of metabolic dysfunction-associated steatohepatitis and may further develop into cirrhosis, liver failure, and even hepatocellular carcinoma in the absence of timely diagnosis and effective intervention [40]. Previous studies have demonstrated a correlation between serum galectin-1 levels and obesity [41] and IR [42]. Nevertheless, the current research exploring the association between galectin-1 and MASLD, which was previously referred to as NAFLD or MAFLD, remains rather limited. In our study, the expression of galectin-1 was increased in the MASLD population. In order to ascertain whether galectin-1 may be implicated in the progression of MASLD, we analyzed the relationship between galectin-1 and MASLD-related metabolic indicators. Derived from BMI, WC, TG, and GGT levels, the FLI serves as a prevalent screening instrument in the clinical and epidemiological assessment of MASLD [43]. The FLI is scored on a scale from 0 to 100. An FLI score of less than 30 suggests a reduced probability of having fatty liver, whereas a score of 60 or above indicates an increased likelihood of the condition [44]. Compared with Western populations, Asian populations exhibit a lower FLI threshold for identifying the presence of hepatic steatosis [45]. The HSI is a simple and effective tool for assessing the severity of hepatic steatosis [34]. Our findings indicate that, after adjusting for gender, age, BMI, and other factors, galectin-1 levels were still positively correlated with the FLI and HSI. If this relationship can be confirmed with a larger cohort, serum galectin-1 levels may be a diagnostic biomarker for hepatic steatosis.

In recent years, there has been increasing support for the important contribution of galectin-1 in adipose tissue homeostasis as a novel adipokine identified by proteomics of subcutaneous adipose tissue interstitial fluid in vivo. Galectin-1 increases in response to weight gain with a high-calorie diet [19]. The oral administration of thiodigalactoside (TDG), an inhibitor of galectin-1, effectively mitigated weight gain and adiposity, along with reducing glucose and triglyceride concentrations in obese rats on a HFD [46]. Jung-Hwan Baek et al. showed that galectin-1 contributes to the progression of obesity in mice on a high-fat diet through the stimulation of the peroxisome proliferator-activated receptor γ (PPARγ) [24]. Analyzing liver transcriptome profiles of pediatric MASLD cohorts with multi-ethnic backgrounds has revealed novel genes associated with disease stages, including galectin-1 [38]. Our research detected a marked elevation in both the protein and mRNA levels of galectin-1 in mouse MASLD models, as compared to the normal control group. This targeted hepatic evidence implies that galectin-1 could be implicated in the pathophysiological processes underlying lipid metabolism dysregulation. These results imply that galectin-1 may be a feasible therapeutic target for MASLD, which can be further studied for clinical application.

There are several limitations that need to be clarified. First of all, we were unable to definitively demonstrate the cause-and-effect relationship between serum galectin-1 levels and the development of MASLD. The second limitation of our research pertains to the use of abdominal color Doppler ultrasound for MASLD diagnosis, which, unlike a liver biopsy, does not enable the quantification of hepatic steatosis or the classification of MASLD stages. Third, the study had a limited sample size, which may have introduced statistical errors. Finally, there are few studies on the dynamic changes in serum galectin-1 levels as the condition evolves.

Future research should prioritize key areas to further elucidate the role of galectin-1 in MASLD and its therapeutic potential. Mechanistic studies are necessary to investigate the specific pathways through which galectin-1 contributes to liver steatosis and inflammation. Longitudinal studies should aim to monitor the temporal changes in galectin-1 levels in MASLD patients, thereby evaluating its prognostic value for disease progression and treatment response. Moreover, research should explore the potential of galectin-1 inhibitors as therapeutic agents for MASLD, building upon existing findings in oncology and other inflammatory disorders. By focusing on these areas, future studies can enhance our understanding of MASLD and facilitate the development of more effective diagnostic and therapeutic strategies.

## 5. Conclusions

In conclusion, this study has identified a significant finding as follows: individuals with MASLD exhibit elevated levels of circulating galectin-1. Moreover, there were notable correlations between serum galectin-1 and markers of liver steatosis, such as the HSI and FLI. These observations suggest that galectin-1 may serve as a promising biomarker or diagnostic tool for detecting MASLD, potentially enabling the earlier identification and management of this condition.

## Figures and Tables

**Table 1:** Baseline information of enrolled participants (*n* = 128). Values are the mean (SD), median (Q1, Q3), or N (%). Groups were compared using a Student’s test (^a^), the Pearson chi-square test (^b^), or the nonparametric Mann–Whitney U test (^c^) (* *p* < 0.05, ** *p* < 0.01, *** *p* < 0.001). BMI, body mass index; WC, waist circumference; HC, hip circumference; WHR, waist-to-hip ratio; TG, triglycerides; AI, atherosclerotic index; TC, total cholesterol; HDL-C, high-density lipoprotein cholesterol; LDL-C, low-density lipoprotein cholesterol; Lp(a), lipoprotein(a); TP, total protein; Alb, albumin; Glb, globulin; ALT, alanine aminotransferase; AST, aspartate aminotransferase; GGT, γ-glutamyl transpeptidase; ALP, alkaline phosphatase; TB, total bile acid; DB, direct bile acids; IDB, indirect bile acids; HSI, hepatic steatosis index; FLI, fatty liver index.

Variables	Control (*n* = 68)	MASLD (*n* = 60)	*p*
Galectin-1 (ng/mL)	85.23 ± 19.96	107.30 ± 23.62	<0.001 ^a^ ***
Male (%)	33 (48.5)	27 (45.0)	0.690 ^b^
Age (years)	40.676 ± 11.914	43.683 ±11.625	0.124 ^a^
Height (cm)	163.00 ± 8.89	160.57 ± 8.18	0.111 ^a^
Weight (kg)	59.85 ± 9.10	69.84 ± 10.90	<0.001 ^a^ ***
BMI (kg/m^2^)	22.48 ± 2.55	27.03 ± 3.22	<0.001 ^a^ ***
WC (cm)	76.63 ± 6.66	88.17 ± 7.54	<0.001 ^a^ ***
HC (cm)	93.13 ± 4.09	98.57 ± 6.77	<0.001 ^a^ ***
WHR	0.82 ± 0.06	0.90 ± 0.06	<0.001 ^a^ ***
TG (mg∗dL^−1^)	95.660 (70.2, 138.2)	148.804 (105.2, 198.4)	<0.001 ^c^ ***
AI	2.56 ± 1.02	3.56 ± 1.31	<0.001 ^a^ ***
TC (mmol/L)	4.78 ± 0.82	5.03 ± 0.98	0.110 ^a^
HDL-C (mmol/L)	1.41 ± 0.32	1.15 ± 0.26	<0.001 ^a^ ***
LDL-C (mmol/L)	2.99 ± 0.71	3.31 ± 0.87	0.029 ^a^ *
Lp(a) (nmol/L)	38.86 ± 30.12	40.56 ± 36.12	0.772 ^a^
TP (g/L)	71.7 ± 3.23	72.6 ± 3.86	0.141 ^a^
Alb (g/L)	47.1 ± 2.11	47.3 ± 2.19	0.625 ^a^
Glb (g/L)	24.5 ± 2.59	25.3 ± 3.42	0.166 ^a^
AST (U/L)	19.52 ± 6.86	24.26 ± 11.50	<0.001 ^a^ ***
ALT (U/L)	13.500 (10.4, 21.1)	24.600 (19.4, 32.7)	0.007 ^c^ **
GGT (U/L)	17.500 (12.0, 26.8)	35.500 (23.0, 61.3)	<0.001 ^c^ ***
ALP (U/L)	70.1 ± 13.9	77.9 ± 17.0	0.005 ^a^ **
TB (umol/L)	13.47 ± 5.72	11.05 ± 4.75	0.011 ^a^ *
DB (umol/L)	5.00 ± 1.75	4.29 ± 1.40	0.012 ^a^ *
IDB (umol/L)	8.47 ± 4.12	6.77 ± 3.58	0.014 ^a^ *
HSI	29.35 ± 4.58	37.05 ± 5.10	<0.001 ^a^ ***
FLI	74.29 ± 20.08	95.66 ± 6.26	<0.001 ^a^ ***

**Table 2:** Cochran–Armitage test for the trend of MASLD frequency in groups with increasing quartile of serum galectin-1 levels. *** *p* < 0.001.

Item	Galectin-1 (ng/mL)				*p* for Trend
	Q1 (≤79.5)	Q2 (79.6–95.8)	Q3 (95.9–110)	Q4 (>110)	
Control	27 (84.4%)	19 (59.4%)	12 (37.5%)	10 (31.2%)	<0.001 ***
MASLD	5 (15.6%)	13 (40.6%)	20 (62.5%)	22 (68.8%)	

**Table 3:** Correlation analysis of serum galectin-1 with other clinical indicators. Correlation coefficients marked with an “a” were calculated using the Pearson test, and correlation coefficients marked with a “b” were calculated using the Spearman test (* *p* < 0.05, ** *p* < 0.01, *** *p* < 0.001). r: regression coefficient. BMI, body mass index; WC, waist circumference; HC, hip circumference; WHR, waist-to-hip ratio; TG, triglycerides; AI, atherosclerotic index; HDL-C, high-density lipoprotein cholesterol; LDL-C, low-density lipoprotein cholesterol; ALT, alanine aminotransferase; AST, aspartate aminotransferase; GGT, γ-glutamyl transpeptidase; ALP, alkaline phosphatase; TB, total bile acid; DB, direct bile acids; IDB, indirect bile acids; HSI, hepatic steatosis index; FLI, fatty liver index.

Item	r	*p*
Weight (kg)	0.245	0.005 ^a^ **
BMI	0.339	<0.001 ^a^ ***
WC (cm)	0.354	<0.001 ^a^ ***
HC (cm)	0.300	<0.001 ^a^ ***
WHR	0.259	0.003 ^a^ **
TG (mg∗dL^−1^)	0.326	<0.001 ^b^ ***
AI	0.361	<0.001 ^a^ ***
HDL-C (mmol/L)	−0.290	<0.001 ^a^ ***
LDL-C (mmol/L)	0.227	0.010 ^a^ *
AST (U/L)	0.110	0.219 ^a^
ALT (U/L)	0.112	0.207 ^b^
GGT (U/L)	0.188	0.034 ^b^ *
ALP (U/L)	0.126	0.155 ^a^
TB (umol/L)	−0.285	0.001 ^a^ **
DB (umol/L)	−0.275	0.002 ^a^ **
IDB (umol/L)	−0.277	0.002 ^a^ **
HSI	0.314	<0.001 ^a^ ***
FLI	0.370	<0.001 ^a^ ***

**Table 4:** Multivariate regressions for the effect of serum galectin-1 level on the FLI and HSI in different models. Crude model: we did not adjust other covariates. Adjusted Model I^a^: we adjusted age and gender. Adjusted Model I^b^: we adjusted age and WHR. Adjusted Model II^a^: we adjusted age, gender, TC, ALT, AST, and ALP. Adjusted Model II^b^: we adjusted age, WHR, TC, ALP, GGT, and TG. HSI, hepatic steatosis index; FLI, fatty liver index.

	FLI (β (95% CI)*p*)		HSI (β (95%CI)*p*)
**Crude Model**	0.370 (0.107, 0.534) <0.001	**Crude Model**	0.314 (0.146, 0.481) <0.001
**Adjusted Model I^a^**	0.362 (0.205, 0.518) <0.001	**Adjusted Model I^b^**	0.228 (0.0845, 0.372) 0.002
**Adjusted Model II^a^**	0.268 (0.123, 0.412) <0.001	**Adjusted Model II^b^**	0.218 (0.0737, 0.363) 0.003

**Table 5:** Results of the binary logit regression analysis. Dependent variable: control or MASLD. McFadden R^2^: 0.492. Cox and Snell R^2^: 0.493. Nagelkerke R^2^: 0.658. r: regression coefficient. SE: standard error. OR (95% CI): odds ratio (95% confidence interval). HSI, hepatic steatosis index; FLI, fatty liver index; /, not applicable.

Item	r	SE	*p*	OR (95% CI )
HSI	0.211	0.069	0.002	1.234 (1.079~1.412)
FLI	0.082	0.035	0.018	1.085 (1.014~1.162)
Galectin-1 (ng/mL)	0.045	0.014	0.002	1.046 (1.017~1.076)
Intercept	−18.747	3.463	/	/

## Data Availability

The data that support the findings of this study are available upon request from the corresponding author. The data are not publicly available due to privacy or ethical restrictions.

## Supplementary Materials

The following supporting information can be downloaded at: https://www.mdpi.com/article/10.3390/biomedicines13020425/s1, Table S1: Test of normality; Figure S1: Volcano plot analysis of differential gene sets between pediatric control and MASLD; Table S2.1: Collinearity Diagnostics Table (VIF) for FLI (Adjusted Model II^a^: we adjusted age, gender, TC, ALT, AST, ALP); Table S2.2: Collinearity Diagnostics Table (VIF) for HSI (Adjusted Model II^b^: we adjusted age, WHR, TC, ALP, GGT, TG); Table S3.1: The corresponding AUC value of indicators; Table S3.2: Delong test for pairwise comparisons of the AUCs; Figure S2: Representative hematoxylin and eosin (HE)-stained liver sections from control and MASLD model groups in mice.
