# Supplementary material for: Feasibility of Serum Galectin-1 as a Diagnostic Biomarker for Metabolic Dysfunction-Associated Steatotic Liver Disease: A Study on a Segment of the Chinese Population Using Convenience Sampling
Source: Biomedicines. 2025 Feb 10;13(2):425. doi: 10.3390/biomedicines13020425 (PMC11853191; doi:10.3390/biomedicines13020425)
Supplement: Supplementary file 1 [file biomedicines-13-00425-s001.zip › biomedicines-3411463-supplementary.pdf]

Supplementary materials

Supplementary Table S1. Test of normality

Item	group	n	average value	SD	measure of skewness	kurtosis	Kolmogorov-Smirnov test	
							D value	*p*
Galectin-1(ng/ml)	Total	128	95.577	24.322	0.359	-0.005	0.046	0.771
	Normal	68	85.235	19.959	-0.066	-0.802	0.077	0.463
	NAFLD	60	107.298	23.621	0.437	-0.490	0.096	0.221
Age(years)	Total	128	42.086	11.830	0.163	-0.469	0.077	0.084
	Normal	68	40.676	11.914	0.398	0.151	0.068	0.666
	NAFLD	60	43.683	11.625	-0.092	-0.920	0.107	0.113
Height(cm)	Total	128	161.858	8.620	0.250	-0.369	0.064	0.283
	Normal	68	162.999	8.894	0.365	-0.584	0.093	0.201
	NAFLD	60	160.565	8.179	0.017	-0.335	0.056	0.938
Weight(kg)	Total	128	64.529	11.130	0.615	0.425	0.076	0.093
	Normal	68	59.847	9.098	0.425	-0.361	0.098	0.155
	NAFLD	60	69.835	10.899	0.671	0.407	0.074	0.604
BMI (kg/m2)	Total	128	24.613	3.665	0.479	0.021	0.079	0.067
	Normal	68	22.480	2.546	0.346	-0.348	0.076	0.484
	NAFLD	60	27.031	3.220	0.386	0.287	0.096	0.217
WC (cm)	Total	128	82.039	9.120	0.330	-0.231	0.077	0.085
	Normal	68	76.632	6.658	0.395	-0.271	0.110	0.056
	NAFLD	60	88.167	7.538	0.295	0.353	0.087	0.356
HC (cm)	Total	128	95.680	6.127	0.736	1.049	0.102	0.004**
	Normal	68	93.132	4.088	0.215	-0.133	0.078	0.452
	NAFLD	60	98.567	6.773	0.282	0.570	0.076	0.561

WHR	Total	128	0.857	0.069	0.207	-0.526	0.076	0.064
	Normal	68	0.822	0.056	0.405	-0.158	0.082	0.370
	NAFLD	60	0.896	0.063	-0.135	-0.112	0.078	0.515
TG (mg*dL-1)	Total	128	150.853	153.091	5.552	37.780	0.247	< 0.001***
	Normal	68	111.955	62.878	2.723	12.236	0.158	< 0.001***
	NAFLD	60	194.937	205.522	4.336	21.031	0.317	< 0.001***
AI	Total	128	3.032	1.264	1.367	3.492	0.119	< 0.001***
	Normal	68	2.563	1.019	1.299	2.125	0.133	0.008**
	NAFLD	60	3.563	1.311	1.477	4.292	0.113	0.070
TC (mmol/L)	Total	128	4.896	0.904	0.588	0.847	0.047	0.742
	Normal	68	4.776	0.818	0.850	2.257	0.087	0.288
	NAFLD	60	5.032	0.983	0.313	0.157	0.060	0.884
HDL-C(mmol/L)	Total	128	1.287	0.320	0.354	-0.688	0.100	0.006**
	Normal	68	1.409	0.320	-0.132	-1.007	0.100	0.133
	NAFLD	60	1.150	0.261	0.923	1.759	0.107	0.108
LDL-C(mmol/L)	Total	128	3.140	0.802	0.464	0.931	0.056	0.483
	Normal	68	2.993	0.714	1.240	4.687	0.093	0.202
	NAFLD	60	3.306	0.868	-0.177	-0.316	0.064	0.813
lip (a) (nmol/L)	Total	128	39.657	32.949	1.133	0.655	0.140	< 0.001***
	Normal	68	38.859	30.123	0.907	0.265	0.111	0.055
	NAFLD	60	40.561	36.124	1.261	0.728	0.212	< 0.001***
TP (g/L)	Total	128	72.107	3.554	0.050	0.550	0.040	0.915

	Normal	68	71.672	3.230	-0.405	-0.232	0.062	0.787
	NAFLD	60	72.601	3.857	0.239	0.696	0.085	0.388
Alb (g/L)	Total	128	47.220	2.143	0.081	-0.270	0.045	0.796
	Normal	68	47.132	2.114	0.351	0.146	0.082	0.376
	NAFLD	60	47.319	2.189	-0.203	-0.518	0.065	0.798
Glb (g/L)	Total	128	24.888	3.021	0.578	1.119	0.047	0.766
	Normal	68	24.540	2.591	0.143	-0.255	0.047	0.978
	NAFLD	60	25.282	3.424	0.646	1.107	0.083	0.430
AST (U/L)	Total	128	21.743	9.587	2.893	10.691	0.209	< 0.001***
	Normal	68	19.525	6.859	2.534	8.502	0.189	< 0.001***
	NAFLD	60	24.258	11.502	2.620	7.879	0.244	< 0.001***
ALT (U/L)	Total	128	23.991	18.777	2.580	7.819	0.206	< 0.001***
	Normal	68	17.734	13.975	3.452	15.748	0.190	< 0.001***
	NAFLD	60	31.082	20.998	2.292	5.501	0.235	< 0.001***
GGT (U/L)	Total	128	38.103	44.342	3.164	11.518	0.273	< 0.001***
	Normal	68	26.838	38.608	4.446	20.783	0.339	< 0.001***
	NAFLD	60	50.869	47.198	2.695	9.296	0.211	< 0.001***
ALP (U/L)	Total	128	73.747	15.840	0.241	-0.480	0.053	0.560
	Normal	68	70.059	13.886	0.451	0.342	0.070	0.629

	NAFLD	60	77.927	16.960	-0.107	-0.748	0.077	0.541
TB (μmol/L)	Total	128	12.339	5.405	1.610	4.157	0.117	< 0.001***
	Normal	68	13.474	5.719	1.728	4.715	0.140	0.004**
	NAFLD	60	11.053	4.752	1.384	2.384	0.154	0.002**
DB (μmol/L)	Total	128	4.668	1.628	1.186	2.685	0.102	0.004**
	Normal	68	5.004	1.750	1.215	2.743	0.124	0.019**
	NAFLD	60	4.288	1.397	0.916	1.421	0.175	< 0.001***
IDB (μmol/L)	Total	128	7.671	3.951	1.577	4.229	0.124	< 0.001***
	Normal	68	8.469	4.115	1.829	5.030	0.142	0.003**
	NAFLD	60	6.766	3.578	1.231	2.456	0.141	0.007**
HSI	Total	128	32.961	6.169	0.305	-0.615	0.094	0.013*
	Normal	68	29.353	4.585	0.754	0.244	0.147	0.002**
	NAFLD	60	37.049	5.104	-0.037	0.157	0.069	0.729
FLI	Total	128	84.308	18.589	-1.345	0.720	0.201	< 0.001***
	Normal	68	74.287	20.077	-0.627	-0.861	0.160	< 0.001***
	NAFLD	60	95.664	6.260	-2.610	7.459	0.246	< 0.001***

* $p < 0.05$, ** $p < 0.01$, ***$p < 0.001$

The normality test is strict and difficult to meet. If the absolute value of kurtosis is less than 10 and the absolute value of skewness is less than 3, it means that the data is not absolutely normal, but can be basically accepted as normal distribution. In conclusion: Different groups of samples for galectin-1, Age, Height, Weight, BMI, WC, HC, WHR, AI, TC, HDL-C, LDL-C, Lip(a), TP, Alb, Glb, TB, DB,

IDB, ALT, ALP, FLI, and HSI can meet or basically accepted as normal distribution. However, TG, AST, and GGT cannot be considered to satisfy a normal distribution.

Supplementary Figure S1

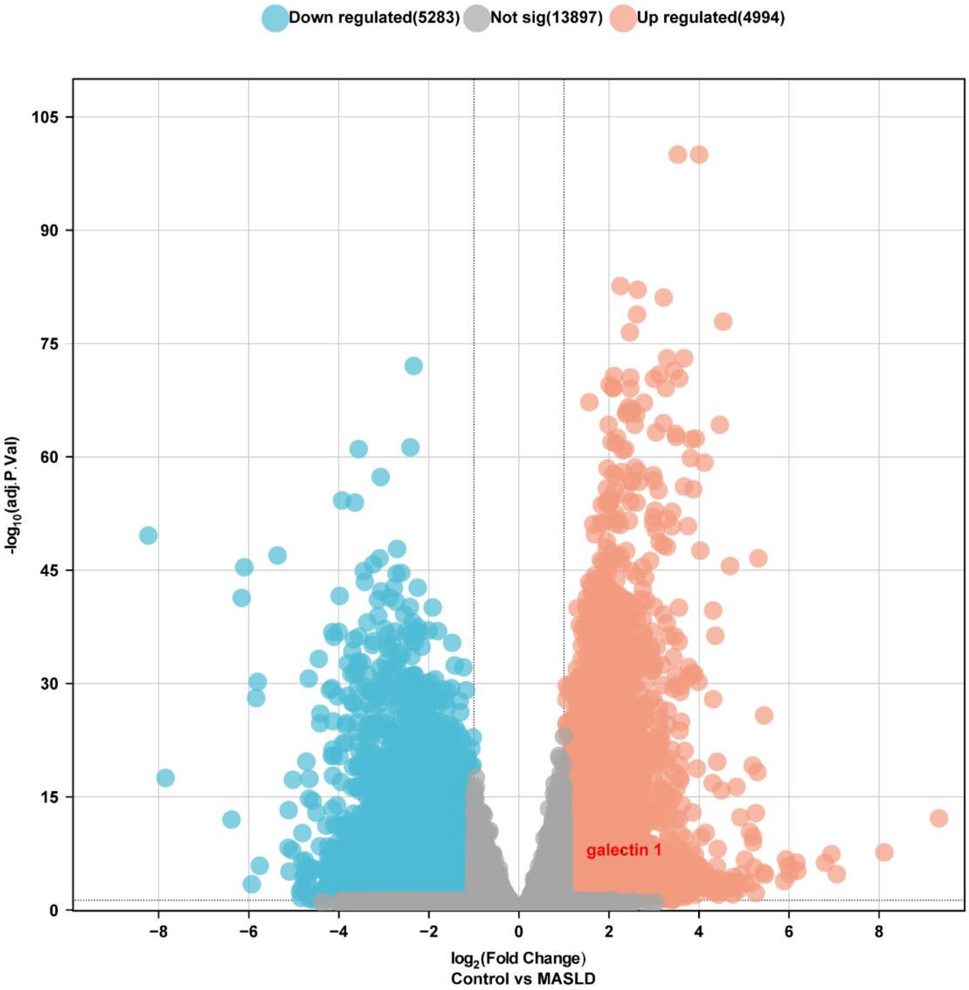

Supplementary Figure S1. Volcano plot analysis of differential gene sets between pediatric Control and MASLD.

Supplementary Table S2.1 Collinearity Diagnostics Table (VIF) for FLI (Adjusted Model II [a]: we adjusted age, gender, TC, ALT, AST, ALP)

Item	VIF
Age(years)	1.372
Gender	1.185
Total cholesterol(mmol/L)	1.180
Alanine aminotransferase(U/L)	2.602
Aspartate aminotransferase(U/L)	2.486
Alkaline phosphatase(U/L)	1.082
Galectin-1(ng/ml)	1.111

D-W value: 2.376 (automatically provided by SPSSAU when performing linear regression)

VIF < 5 , indicating no multicollinearity issues.

Supplementary Table S2.2 Collinearity Diagnostics Table (VIF) for HSI (Adjusted Model II[b]: we adjusted age, WHR, TC, ALP, GGT, TG)

Item	VIF	
Age(years)	1.246	1.246
WHR	1.291	1.291
Total cholesterol(mmol/L)	1.224	1.224
Alkaline phosphatase(U/L)	1.179	1.179
γ-glutamyl transpeptidase(U/L)	1.383	1.383
Triglyceride (mg*dL-1)	1.353	1.353
Galectin-1(ng/ml)	1.160	1.160

D-W value: 1.641 (automatically provided by SPSSAU when performing linear regression)

VIF < 5 , indicating no multicollinearity issues.

Supplementary Table S3

Supplementary Table S3.1 The corresponding AUC value of indicators

Item	AUC	SE	*p*	95% CI
Galectin-1(ng/ml)	0.745	0.043	< 0.001***	0.662 ~ 0.829
HSI	0.869	0.032	< 0.001***	0.806 ~ 0.932
FLI	0.889	0.028	< 0.001***	0.833 ~ 0.944
Combined diagnosis	0.923	0.023	< 0.001***	0.877 ~ 0.969

* $p < 0.05$, ** $p < 0.01$, *** $p < 0.001$

Supplementary Table S3.2 Delong test for pairwise comparisons of the AUCs

Item 1	Item 2	Difference in AUC	SE	95% CI	z	p
Combined diagnosis	Galectin-1(ng/ml)	0.1775	0.0398	0.100 ~ 0.255	4.4630	p< 0.001***
Combined diagnosis	HSI	0.0534	0.0212	0.012 ~ 0.095	2.5192	0.0118*
Combined diagnosis	FLI	0.0338	0.0219	-0.009 ~ 0.077	1.5471	0.1218

* $p < 0.05$, ** $p < 0.01$, *** $p < 0.001$

Supplementary Figure S2

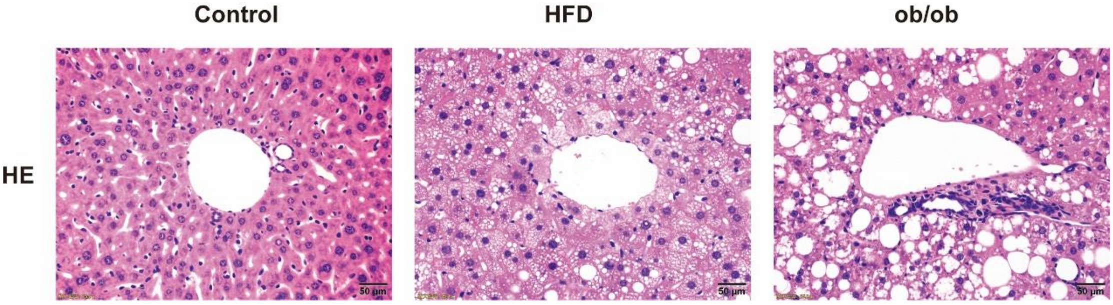

Supplementary Figure S2. Representative hematoxylin and eosin (HE)-stained liver sections from control and MASLD model groups in mice. Scale bars: 50 μm.
